# Time to focus again on matrix metalloproteinases? Results of complex network analysis involving the pathophysiology of HER2-positive breast cancer

**DOI:** 10.3332/ecancer.2025.1850

**Published:** 2025-02-18

**Authors:** Pedro G Buiar, José Danilo Szezech Junior, Matheus Rolim Sales, Giovani Marino Favero

**Affiliations:** 1 Medical Oncology Department, Instituto Sul Paranaense de Oncologia, Ponta Grossa, Brazil; 2 Department of Mathematics and Statistics, State University of Ponta Grossa, Ponta Grossa, PR, Brazil; 3 Biological and Health Science Multidisciplinary Laboratory, State University of Ponta Grossa, Ponta Grossa, Brazil; a https://orcid.org/0000-0001-5144-1197; b https://orcid.org/0000-0001-8306-8315; c https://orcid.org/0000-0002-1121-6371; d https://orcid.org/0000-0002-1946-3262

**Keywords:** breast cancer, complex network, HER2, metalloproteases, antibody conjugate

## Abstract

Breast cancer is the most common cancer in women worldwide, with significant advances in understanding its multifactorial nature in recent years. The complex structure of molecular and cellular interactions in cancer pathophysiology presents challenges for developing effective treatments. One theoretical model used to study these interactions is the Graph model or Complex Networks, which uses mathematical methods to create graphical figures by connecting vertices (factors) through edges (interactions). This study uses the graph model to determine the complex interactions within the tumour microenvironment of HER2-positive breast cancer. Through a narrative review, 37 factors involved in the pathophysiology of HER2-positive breast cancer were identified and incorporated into a complex network design, starting with the HER2 vertex. The impact of each vertex was determined by calculating the relative error, and a knockout (KO) analysis of vertices was performed to identify their influences within the network. The Wilcoxon test was used to analyze the statistical significance of each KO. Significant alterations in the network structure were observed with the KOs of matrix metalloproteinases (MMPMMP2, MMP9, cyclin-dependent kinases 4/6, TWIST, vascular endothelial growth factor and transforming growth factor-beta. Notably, the KOs of (MMPs) MMP2 and MMP9 significantly impacted the network structure and downregulated the HER2 vertex. This raises questions about the potential applicability of targeting MMPs, including the option of HER2-directed antibody-drug conjugates. Could a metalloprotease inhibitor be a good choice for conjugation? Despite the theoretical nature of this model, the results suggest potential avenues for therapeutic intervention.

## Background

Breast cancer with human epidermal growth factor receptor 2 (HER-2) expression is a distinct subtype characterised by features that differentiate it from other forms of breast cancer. The pathophysiology of this tumour involves a higher potential for replication and carcinogenesis [1]. Recent advances in oncology targeting the HER-2 receptor have significantly improved prognosis and cure rates [2]. However, the emergence of resistance mechanisms to HER-2 blockade is common [3]. This resistance arises from a complex pathophysiology involving multiple genetic, molecular and cellular factors within the tumour microenvironment. Identifying which factors are directly or indirectly associated with the therapeutic target presents a challenge for oncology researchers, but it also holds the key to therapeutic advancements.

To explore the intricate interactions within complex systems, physics has developed the theory of complex networks, also known as graph theory. These networks are analytical tools that, through abstraction, model interactions between elements. This is achieved by constructing an adjacency matrix. This mathematical model not only facilitates the analysis of the connections and the intriguing structure of the network but also enables the generation of hypotheses and the derivation of conclusions, such as the relative importance of each factor within the network.

In oncology, understanding the mechanisms underlying tumour resistance to treatments is crucial. Among potential therapeutic drugs under development, the vast majority fail to demonstrate efficacy due to the multifactorial and complex pathophysiology of cancer. In this study, we aim to identify, using a complex network model, the molecular and cellular factors most critical in the pathophysiology of HER-2-positive breast tumours [3]. Our hypothesis is that applying a complex network model could help elucidate potential points of tumour resistance to current treatments, thereby identifying new therapeutic targets and synergistic therapeutic combinations. This approach exemplifies the application of mathematical models in oncology.

## Methods

### Selection of molecular and cellular factors to be studied

An extensive narrative literature review on the pathophysiology of breast cancer was conducted using the PubMed and Google Scholar platforms. The MeSH terms [Breast Cancer] AND [HER-2] were initially used as a starting point. The search results were selected after evaluating the title and abstract for the association of the searched terms. At least ten articles were selected and analyzed in full. As a result of this initial search, other molecular and cellular factors interacting with HER2 in breast cancer were identified. For each of these factors, a new search was conducted on the platforms using [Breast Cancer] AND [factor xxx]. This methodology was followed, selecting at least ten articles for each molecular factor, preferably review articles and excluding articles that did not involve breast cancer. The predetermined number of factors to compose the complex network to be created was set at 37 objects. Once this number was reached, the literature search was terminated. The 37 chosen factors were HER-2, Rat Sarcoma Virus protein (RAS), Phosphoinositide 3-kinase (PI3K), Proto-oncogene Src, Extracellular-signal regulated kinase (ERK), mammalian target of Rapamycin (mTOR), insulin-like growth factor 1 receptor (IGF1), proto-oncogene MYC, estrogen receptor (ER), proline, glutamate and leucine rich protein 1 (PELP1), androgen receptor (AR), MMP2 and MMP9 (Matrix Metalloproteinase (MMPs)), Twist-related Protein 1, Interleukin-6 (IL-6), Janus Kinase Factor (JAK), signal transducer activator of transcription 3 (STAT3), N-cadherin, vascular endothelial growth factor (VEGF), fibroblast growth factor 2 (FGF2), platelet-derived growth factor (PDGF), SNAIL protein, Wnt (Wingless gene), CCL-2 (The Chemokine C-C Motif Ligand 2), cyclin dependent kinases (CDK 4/6), stem cell factor (SCF), transforming growth factor-beta (TGF-β), tumour necrosis factor alpha (TNF-alpha), nuclear factor kappa-light-chain-enhancer of Activated B Cells (NF-kB), T Lymphocytes, Neutrophils, Cancer-associated Fibroblasts, Cancer-associated Macrophages, intra-tumoural Mast Cells, cancer stem cells (CSCs), TAZ signal transducer, Cancer-associated Adipocytes and Tissue Hypoxia. After reading the selected articles, each factor was analyzed separately, and those other network components with which the factor in question has a positive interaction, i.e., an agonistic stimulus, were identified.

### The dynamical stochastic model

**Figure 1.**
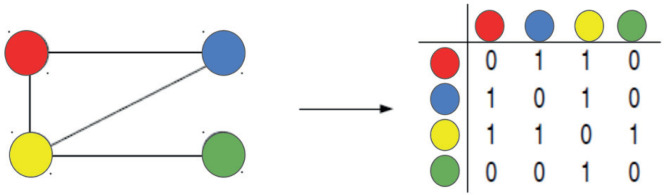
Adjacency matrix represents the interactions among four distinct vertices of a network.

A complex network [5–8] is defined as a set of *V* nodes or vertices, and *L* links or edges. The topology of the connections is represented by the adjacency or connectivity, matrix *A* = {*aij*}, where *i*, *j* = 1, 2,…, *V*, with elements equal to 1 if the node *j* is connected to the node *i* and 0 otherwise (illustrated in Figure 1).

If the connections are directed *aij* ≠ *aji*, in general. The complex network is considered a fixed network, with a constant number of vertices and edges. The network is also complete, meaning there are no unconnected vertices on the graph [5]. There are several measures to characterise the structure of a complex network. We will focus on the simplest measure: the degree, that characterises the connectivity properties of a single vertex in a complex network. We define two-degree measures for each vertex, the in-degree, *k_i,_*_in_, and the out-degree, *k_i_*_,out_, respectively, as follows.

These measures simply count the number of incoming (in-degree) and outgoing (out-degree) connections of a vertex. In a connected network, *k_i_* ≠ 0 for all vertices.

The vertices in the network interact dynamically through the connections given by the adjacency matrix. To model these dynamics, we define a random walk on the network. The random walk is a stochastic process in discrete time steps, in which a walker follows a path defined by the network topology.

A random walk is a simple mathematical process in which a 'walker' moves step by step from one point to another, with the direction of each step chosen randomly. Similar to a person navigating a city and selecting a random street at each intersection without a predetermined destination, the random walk represents a stochastic process occurring in discrete time steps, where the path is dictated by the topology of the network. In the context of a complex network, composed of numerous interconnected nodes (analogous to cities on a map), the walker is a conceptual entity that traverses between these nodes. At each step, the walker randomly selects one of the adjacent nodes to move to. Over time, the walker explores the network by randomly visiting different nodes. Random walks are particularly useful for studying the spread of information or entities, the degree of connectivity within the network and the ease with which movement occurs from one region to another. This approach is valuable in understanding various types of networks, such as social media, biological systems and the Internet.

In our model, the walkers depart from a fixed vertex, and the process is given by the following steps:

1. At an initial instant of time *t* (discrete), a walker *w* initially at the vertex *i* = 1 of the network, changes its position to a vertex *j* as time changes from *t* to *t* + 1.
2. The walker shifts its position randomly respecting the directionality of the edges, i.e., the walker is allowed to shift from vertex *i* to vertex *j* if, and only if, *a_ij_* = 1.
3. As time changes from *t* to *t* + 1, a new walker is created at the vertex *i* = 1, and the positions of all walkers are updated according to steps 1 and 2.

The total number of walkers on the network is *N*(*t*) = *t* and the concentration of walkers on each vertex at an instant of time *t* is given by the vector ***S***(*g*; *t*) = (*s*_1_(*t*), *s*_2_(*t*),…, *sv*(*t*))

***S***(*g*; *t*) = (*s*_1_(*t*), *s*_2_(*t*),…, *sv*(*t*))

where *si*(*t*) corresponds to the total number of walkers on the *i*th vertex at the instant of time *t*. Therefore, the density or flux, of walkers of each vertex is and the flux vector is defined as follows:

**f**(*g*; *t*) =(*f*_1_(*t*), *f*_2_(*t*),…, *fv*(*t*))

The larger the values of *f_i_*(*t*), the more the vertex *i* is being activated, or the more information is passing through this agent. Since the network is connected, the flux vector of walkers, **f**, tends to a stationary distribution as *t* → ∞.

To understand how the topological changes in the network influence the statistical behavior of **f**, we knock out (KO) a vertex, i.e., remove it from the network, resulting in a new graph *g*′, and we study the quantity Δ**f** = **f**(*g*; *T*) − **f**(*g*′; *T*)

where **f**(*g*′; *T* ) corresponds to the flux vector on the KO network. The elements of Δ**f** can be either positive or negative. If positive (negative), it means the KO increases (decreases) the activation of the agent *i*. The relative error of each vertex is defined as follows:

Note that dim(***μ***) = dim(Δ**f**) = *V* − 1. The average of the relative error is simply where *i*KO corresponds to the index of the KO vertex.

To numerically obtain the flux of walkers, we consider a total time of *T* = 10^4^, where in each time step a new walker is created, resulting in 10^4^ walkers. We repeat this procedure *L* = 100 times and compute the mean flux vector as follows:

We KO each vertex in the network, except for vertex 1, which serves as the starting vertex, and compute the mean flux vector for each KO network. Using the mean flux vector of the healthy network and the mean flux vector of all KO networks, we calculate the mean relative error, *ū_i_*, which is the relative error of the mean distributions, and calculate the average relative error *M*(*g*, *g*′; *T*,*L*).

For the statistical analysis, we perform a Wilcoxon signed rank test to compare the healthy network with each KO network, using a p-value <0.05 as the threshold for statistical significance.

To construct the network we used the Python programming language and employed resources from the Igraph package, along with the NumPy package to perform the calculations.

## Results

**Figure 2.**
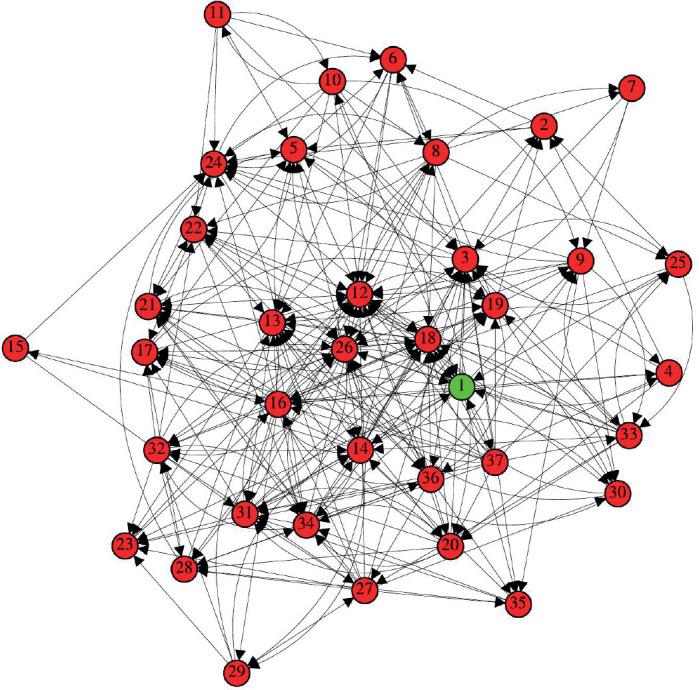
Network of immunological interactions for. The vertices labels are: 1 – HER-2, 2 – RAS, 3 – PI3K, 4 – Src, 5 – ERK, 6 – mTOR, 7 – IGF1, 8 – MYC, 9 – ER, 10 – PELP1, 11 – AR, 12 – MMP2 and MMP9, 13 – TWIST, 14 – IL-6, 15 – JAK, 16 – STAT3, 17 – N-cadherin, 18 – VEGF, 19 – FGF2, 20 – PDGF, 21 – SNAIL, 22 – WNT, 23 – CCL2, 24 – CDK4/6, 25 – SCF, 26 – TGF-B, 27 – TNF-α, 28 – NF-kB, 29 – T lymphocytes, 30 – Neutrophils, 31 – Fibroblasts, 32 – Macrophages, 33 – Intra-tumoural mast cells, 34 – Cancer stem cells, 35 – TAZ signal transducer, 36 – Adipocytes and 37 – Tissue hypoxia.

After processing the complex network, the graph shown in Figure 2 was generated.

### Analysis of the degree of each vertex

In a visual analysis of the graph, we can quickly see that some vertices concentrate a greater number of inputs and outputs, drawing attention due to their degree and density within the network.

To obtain the numerical values of the walker flows f*_i_*(t), we considered a total time of T = 10^4^, where at each time step a new walker is created, resulting in a total of 10^4^ walkers by the end of the simulation. We repeated the procedure L = 100 times, and at the end, we took the average of the vector f(g; T), that is,

where f*_i_*(g; T) corresponds to the stationary distribution obtained for T = 104.

**Figure 3.**
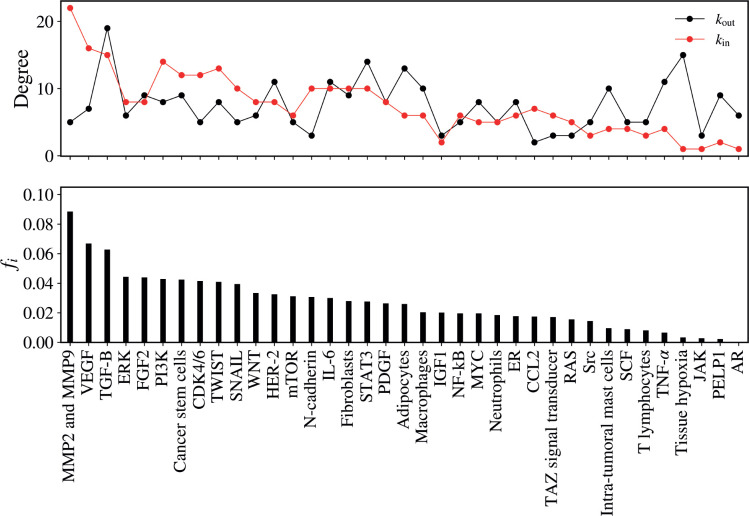
(Top) In-degree (red) and the out-degree (black) for the vertices. (Bottom) the mean flux of walkers, fi, for the same vertices.

The average flow of walkers per vertex, including both input and output for each vertex of the network, is illustrated in Figure 3. In this figure, the vertices on the horizontal axis are ordered in descending order according to the value of the flow *f_i_*.

The vertex that presented the highest flow of walkers was MMPs MMP2 and MMP9 (*f_i_*= 0.08845), followed by VEGF (*f_i_* = 0.06685), TGF-B (*f_i_* = 0.06270), ERK (*f_i_* = 0.04431), CDK4/6 (*f_i_* = 0.04145), CSC (*f_i_* = 0.04241), TWIST (*f_i_* = 0.04087), SNAIL (*f_i_* = 0.03938), PIK3K (*f_i_* = 0.04278) and STAT3 (*f_i_* = 0.02760), as illustrated in Table 1.

### Vertex KO

A KO analysis was performed on each vertex, and the impact of each KO was calculated using the relative error. The six vertices that showed the highest relative errors and had the most significant impact on the network were MMP2 and MMP9 (0.19943), VEGF (0.18514), PI3K (0.16268), TGF-B (0.15465), CDK4/6 (0.12628) and TWIST (0.12596). For comparison between the ‘healthy network’ and the ‘experimental network with KO’, a statistical analysis was performed using the Wilcoxon signed-rank nonparametric test for paired numerical variables. The Wilcoxon Rank-Sum Test is a non-parametric statistical test used to compare two independent groups to determine whether one group tends to have higher or lower values than the other. We use it as an alternative to the *t*-test, assuming that the data extracted from a complex network does not follow a normal distribution. Interpreting the KO analysis as a scenario where a potential treatment or therapeutic blockade silences a target vertex, the Wilcoxon test is appropriate for comparing the effectiveness of two treatments when the data are skewed. The values plotted in the comparative test between the two groups (the healthy network and the KO network) represent the 'mean flux of walkers' for each vertex. We present the results of the KO analysis for the six KOs with the highest average relative errors: MMP2 and MMP9, VEGF, PI3K, TGF-β, CDK4/6 and TWIST.

### MMP-2 e MMP-9 KO

**Figure 4.**
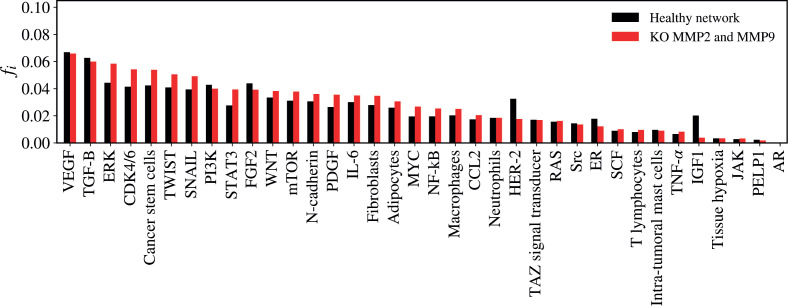
Mean flux of walkers, *f(i)*, for the healthy network (black) and the KO MMP2 and MMP9 (red).

The KO of MMP2 and MMP9 significantly impacted the network structure (*Z* = −2.443; *p* = 0.01468), altering the flow of inputs and outputs of the other vertices (Figure 4). The five vertices with the largest variations in walker flow caused by the KO of MMP2 and MMP9 were IGF-1 (Δ*f_i_* = −0.01631); HER-2 (Δ*f_i_* = −0.01492); ERK (Δ*f_i_* = 0.01411); CDK4/6 (Δ*f_i_* = 0.01276); STAT3 (Δ*f_i_* = 0.01176).

### VEGF KO

**Figure 5.**
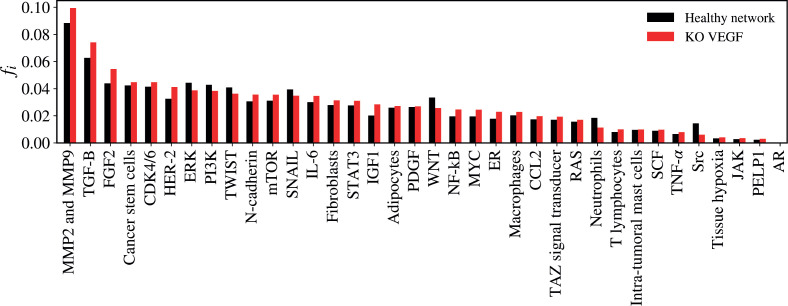
Mean flux of walkers, *f(i)*, for the healthy network (black) and the KO VEGF (red).

The KO of VEGF resulted in a significant alteration in the network (*Z* = −2.3723; *p* = 0.01778) as demonstrated in Figure 5. The five vertices with the greatest difference following the KO of VEGF were TGF-B (Δ*f_i_* = 0.01147); MMP2 and MMP9 (Δ*f_i_* = 0.01106); FGF2 (Δ*f_i_* = 0.01062); HER-2 (Δ*f_i_* = 0.00872); Src (Δ*f_i_* = −0.00839).

### PI3K KO

**Figure 6.**
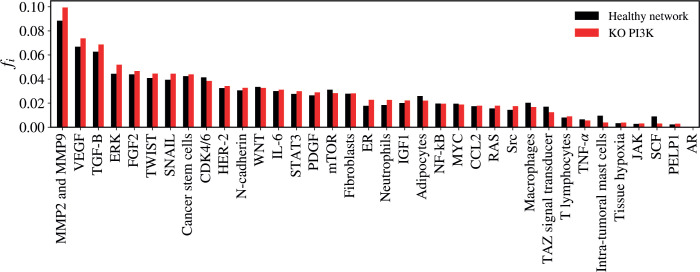
Mean flux of walkers, *f(i)*, for the healthy network (black) and the KO PI3K (red).

The KO of PI3K resulted in a non-significant alteration in the network (*Z* = −1.8774; *p* = 0.0601). The five vertices with the greatest difference following the KO of PI3K were MMP2 and MMP9 (Δ*f_i_* = 0.01095); ERK (Δ*f_i_* = 0.00764); VEGF (Δ*f_i_* = 0.00692); TGF-B (Δ*f_i_* = 0,00601); SCF (Δ*f_i_* = - 0.00592), as verified in Figure 6.

### TGF-B KO

**Figure 7.**
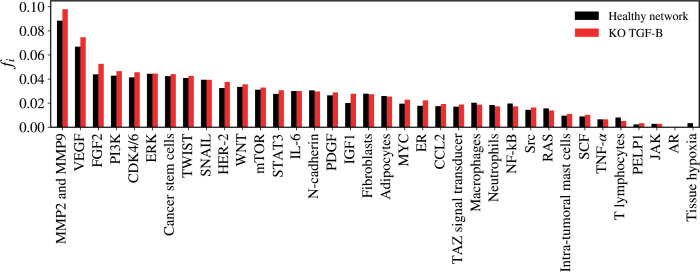
Mean flux of walkers, *f(i)*, for the healthy network (black) and the KO TGF-B (red).

The KO of TGF-B resulted in a statistically significant alteration in the network (*Z* = −2.981; *p* = 0.00288) as demonstrated in Figure 7. The five vertices with the greatest difference following the KO of TGF-B were MMP2 and MMP9 (Δ*f_i_* = 0.00955); FGF-2 (Δ*f_i_* = 0.00872); VEGF (Δ*f_i_* = 0.00787); IGF-1 (Δ*f_i_* = 0.00765); HER-2 (Δ*f_i_* = 0.00513).

### CDK4/6 KO

**Figure 8.**
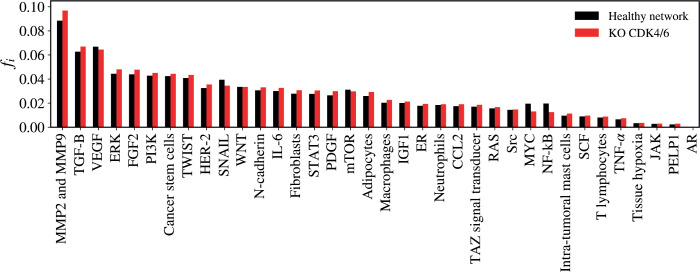
Mean flux of walkers, *f(i)*, for the healthy network (black) and the KO CDK4/6 (red).

The KO of CDK4/6 resulted in a statistically significant alteration in the network (*Z* = −3.1028; *p* = 0.00194) as demonstrated in Figure 8. The five vertices with the greatest difference following the KO of CDK4/6 were MMP2 and MMP9 (Δ*f_i_* = 0.00845); NF-kB (Δ*f_i_* = −0.00707); MYC (Δ*f_i_* = −0.00643); SNAIL (Δ*f_i_* = −0.00499); FGF2 (Δ*f_i_* = 0.00389).

### TWIST KO

**Figure 9.**
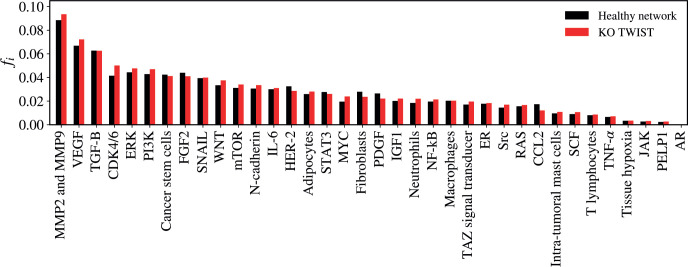
Mean flux of walkers, *f(i)*, for the healthy network (black) and the KO TWIST (red).

The KO of TWIST resulted in a statistically significant alteration in the network (*Z* = −2.4508; *p* = 0.01428) as demonstrated in Figure 9. The five vertices with the greatest difference following the KO of TWIST were CDK4/6 (Δ*f_i_* = 0.00868); VEGF (Δ*f_i_* = 0.00541); CCL2 (Δ*f_i_* = −0.00533); MMP2 and MMP9 (Δ*f_i_* = 0.00513); MYC (Δ*f_i_* = 0.00446).

## Discussion

Up to 15% of breast cancer cases present with HER-2 overexpression [9]. The HER-2 receptor is a transmembrane receptor belonging to the epidermal growth factor receptor family. When activated, it triggers cancerous cellular machinery through molecular cascades, including growth pathways, cell proliferation, angiogenesis and others [10]. One of the most significant advances in oncology involved the treatment of breast cancer by targeting the HER-2 receptor, initially through the antibody trastuzumab. In 2001, robust data demonstrated that adding HER-2 receptor blockade to chemotherapy prolonged the median overall survival of patients with metastatic breast cancer by approximately 5 months [11]. These results were quickly replicated in the adjuvant setting, leading to cures for patients after surgery, with absolute gains of 8%–9% in progression-free survival and overall survival rates at 5 years [12–14]. This progress was further enhanced by the introduction of dual HER-2 receptor blockade with pertuzumab and trastuzumab, which radically improved the prognosis and course of the disease in both the adjuvant and metastatic settings [15, 16].

The central role of HER-2 in the pathophysiology of this breast cancer subtype was confirmed by the significant impact of HER-2 blockade on response rates. This is why HER-2 was chosen as the starting point for exploring network interactions. However, cancer pathophysiology is not dependent on a single pathway; cancer can progress through various mechanisms. Along the spectrum of breast cancer aggressiveness, we find the triple-negative subtype at one extreme and the luminal A subtype at the other, with HER-2-overexpressing tumours often situated between these points. These tumours frequently co-express estrogen and progesterone hormone receptors. While HER-2 blockade has had a transformative impact on this cancer subtype, the advent of CDK-cyclin inhibitors (iCDK) in the luminal subtype has also significantly improved survival in patients with hormone receptor-positive breast cancer [17–19]. However, the combination of iCDK with trastuzumab in HER-2-positive disease has not demonstrated superior outcomes compared to standard chemotherapy combined with trastuzumab [20]. Additionally, evidence suggests that patients treated with iCDK whose tumours had a HER-2 score of 0 responded better than those with low HER-2 expression [21, 22]. In contrast to the triple-negative scenario, where recent advances with immune checkpoint inhibitors have established immunotherapy as a viable strategy in both the neoadjuvant and metastatic settings, the addition of immunotherapy to HER-2-positive breast cancer has not yielded satisfactory or statistically significant results [23, 24].

Our complex network model, which involves potential molecular and cellular factors in the pathogenesis of breast cancer, indicated that the most impactful factors on the network as a whole were MMP2 and MMP9, with statistically significant results (*p* = 0.01468). Studies have highlighted the role of these molecules in breast cancer, pointing to their higher expression in the extracellular matrix of the tumour microenvironment compared to adjacent normal tissue. Additionally, elevated levels of MMP2 and MMP9 are associated with worse prognosis, greater lymph node dissemination and increased tumour aggressiveness [25, 26]. When these findings are combined with the primary outcome of our mathematical model, MMP2 and MMP9 emerge as potential therapeutic targets for combating breast cancer.

MMPs are zinc-dependent endopeptidases produced by both tumour and non-tumour cells within the tumour microenvironment, with MMP2 and MMP9 classified as gelatinases [27]. These enzymes contribute to tumour progression, pro-tumour factor production, angiogenesis and metastasis by remodeling and degrading the extracellular matrix [28]. In our model, the KO of MMP2 and MMP9 significantly altered the network structure, resulting in a marked reduction in the flow of walkers passing through the HER2 vertex. This effect was similar to the KO of TWIST but more pronounced. Conversely, the KO of VEGF, TGF-β, CDK4/6 and PI3K led to an increase in the influx of walkers passing through HER2, potentially stimulating this vertex. These findings suggest that inhibiting MMP2 and MMP9 could influence the tumour biology of HER2-overexpressing breast cancer.

Several molecules with potential enzymatic inhibitory effects on MMPs have been developed in laboratory settings. Some MMP9 inhibitors have undergone testing in various cancers during phase I–III clinical trials. While these trials have demonstrated antitumour activity, they have not shown significant clinical superiority or were terminated due to issues such as poor oral bioavailability and significant adverse effects [29]. In the context of breast cancer specifically, Marimastat was tested in a phase III clinical trial involving patients with metastatic breast cancer who had shown a clinical response or disease stability after first-line chemotherapy. No difference in progression-free survival was observed compared to placebo, and an increase in adverse event rates was reported [30]. It is noteworthy that patients in this study were not stratified based on HER2 expression. Another drug, BMS-275291, was tested as an adjuvant treatment for breast cancer patients in clinical stages I–III. However, the trial was prematurely terminated due to side effects [31]. Subsequently, trials with these drugs were suspended, and various strategies to modify molecular composition to improve selectivity are currently being explored [32].

The results of this complex network model highlight the importance of exploring alternative approaches to target the enzymatic activity of tumour-associated MMP2 and MMP9. One potential mechanism to enhance the effectiveness of such therapies while reducing systemic side effects is combining an MMP enzymatic inhibitor with an HER2-targeting antibody. This hypothesis is supported by recent advances in HER2-positive breast cancer treatments with medications such as trastuzumab-emtansine and trastuzumab-deruxtecan – both drug-antibody conjugates – which have demonstrated significant improvements in tumour response rates and survival outcomes [32, 33].

Various studies have demonstrated methods to link monoclonal antibodies to therapeutic molecules through different mechanisms, including cytotoxic chemotherapeutic agents and target-specific enzyme inhibitors [34, 35].

Accordingly, our complex network model of HER2-overexpressing breast cancer suggests the therapeutic potential of inhibiting MMP2 and MMP9 as a strategy to target tumours. Based on this model, we hypothesise a novel mechanism of action: the development of a conjugate between anti-HER2 antibodies and an MMP2/MMP9 inhibitory enzyme. By targeting tumour cells through HER2 and releasing the MMP2/MMP9 inhibitor at specific sites within the tumour microenvironment, we could achieve reduced adverse events and enhanced therapeutic impact. This approach is particularly promising given the critical role of metalloproteinases in cancer pathophysiology. Furthermore, in the context of HER2 overexpression, this strategy may yield intensified effects. Silencing MMP2 and MMP9 has been shown to reduce the flow of walkers toward HER2 in our model, which could be interpreted as downregulation of the HER2 pathway.

Despite the extrapolations and potential translational hypotheses of this study, it is important to note that it is a theoretical mathematical model that raises hypotheses. Moreover, complex networks represent a relatively new science that, despite its applicability to biological models, still requires further understanding. The inferences drawn from these networks should be validated through practical experimental methodologies. Another potential limitation of complex network models stems from the unpredictability of biological interactions in real life. While the model facilitates the translation and interpretation of relationships among factors, it often fails to accurately incorporate elements such as the spatial and temporal variability intrinsic to biology. Specifically, within the field of oncology, unforeseen mechanisms of resistance may arise over time or may be present from the outset of an intervention.

Our study began with a narrative review of the literature to select the vertices comprising the complex network. A systematic review could have been conducted, which would have enhanced the quality of factor selection; however, it might have limited the number of vertices or restricted the inclusion of vertices that we wished to observe. This approach allowed us greater freedom in selecting the vertices we wanted to see interacting within the network, albeit at the expense of methodological rigor.

## Conclusion

The complex network model is a mathematical tool that allows for the study of interactions between vertices within a matrix, facilitating abstraction and theorisation about the functioning of a complex system. Breast cancer is the most prevalent neoplasm among women worldwide, and increasing efforts are needed to develop new treatments. In the context of HER-2 positive breast cancer, multiple tumour factors appear to revolve around this molecule. Our network model allowed us to verify that the greatest impact on the network was generated by the KO of MMPs. This impact on the network was simultaneously associated with a reduction in the flow of walkers through the HER-2 vertex.

We cannot ascertain whether this is due to chance based solely on the mathematical model; however, data in the literature regarding the role of metalloproteinases in carcinogenesis and the HER2 pathway in breast cancer lead us to believe in the potential of these findings as a foundation for further research, rather than as definitive clinical conclusions. Practical experimental data are necessary to validate the assumptions of this complex network model. The combination of drugs targeting HER2 and MMP could be achieved through available pharmaceutical technologies to conjugate HER-2 binding antibody with an MMP inhibitory enzyme. Further molecular studies and testing are needed; however, the use of the complex network model for hypothesis generation proves to be useful and potentially applicable in the breast cancer scenario and in oncology in general.

## List of abbreviations

AR, Androgen receptor; CCL-2, The chemokine C-C motif ligand 2; CDK 4/6, Cyclin dependent kinases; CSC, Cancer stem cells; ER, Estrogen receptor; ERK, Extracellular-signal regulated kinase; FGF2, Fibroblast growth factor 2; HER-2, Human epidermal growth factor receptor 2; IGF1, Insulin-like growth factor 1 receptor; IL-6, Interleukin-6; JAK, Janus kinase factor; MMP2 and MMP9, Matrix metalloproteinase 2 and 9; mTOR, Mammalian target of rapamycin; NF-kB, Nuclear factor kappa-light-chain-enhancer of activated B cells; PDGF, Platelet-derived growth factor; PELP1, Proline, glutamate and leucine rich protein 1; PI3K, Phosphoinositide 3-kinase; RAS, Rat Sarcoma Virus protein; SCF, Stem cell factor; STAT3, Signal transducer activator of transcription 3; TGF-β, Transforming growth factor-beta; TNF-alpha, Tumour necrosis factor alpha; TWIST1, Twist-related protein 1; VEGF, Vascular endothelial growth factor; Wnt, Wingless gene.

## Conflicts of interest

The authors have no conflicts of interest to declare.

## Funding

All the authors declare no access to funding support.

## Author contributions

- Study conception: Pedro G Buiar; Giovani M. Favero
- Study design: All authors
- Data collection: Pedro G Buiar; Matheus Rolim Sales
- Analysis and interpretation of results: All authors
- Draft manuscript preparation: All authors

All authors reviewed the results and approved the final version of the manuscript.

## Figures and Tables

**Table 1.** Mean flux of walkers of each component for the healthy network.

MMP2 e MMP9	0.08845	N-cadherin	0.03064	RAS	0.01554
VEGF	0.06685	PDGF	0.02642	Src	0.01441
TGF-B	0.0627	IL-6	0.02996	ER	0.0177
ERK	0.04431	Fibroblasts	0.02785	SCF	0.00893
CDK4/6	0.04145	Adipocytes	0.02591	T lymphocytes	0.00803
CSC	0.04241	MYC	0.01952	Intra-tumoral Mast cells	0.00954
TWIST	0.04087	NF-KB	0.01956	TNF-a	0.00658
SNAIL	0.03938	Macrophages	0.02028	IGF-1	0.02013
PI3K	0.04278	CCL2	0.01739	Tissue Hypoxia	0.0033
STAT3	0.0276	Neutrophils	0.01838	JAK	0.00271
FGF2	0.04389	HER-2	0.03244	PELP1	0.00225
WNT	0.03341	TAZ signal transducer	0.01706	AR	0.00025
mTOR	0.03114				
